# Darwin Core: An Evolving Community-Developed Biodiversity Data Standard

**DOI:** 10.1371/journal.pone.0029715

**Published:** 2012-01-06

**Authors:** John Wieczorek, David Bloom, Robert Guralnick, Stan Blum, Markus Döring, Renato Giovanni, Tim Robertson, David Vieglais

**Affiliations:** 1 University of California, Berkeley, California, United States of America; 2 University of Colorado, Boulder, Colorado, United States of America; 3 California Academy of Sciences, San Francisco, California, United States of America; 4 Global Biodiversity Information Facility, Copenhagen, Denmark; 5 Centro de Referência em Informação Ambiental, Campinas, São Paulo, Brasil; 6 University of Kansas, Lawrence, Kansas, United States of America; University of Vermont, United States of America

## Abstract

Biodiversity data derive from myriad sources stored in various formats on many distinct hardware and software platforms. An essential step towards understanding global patterns of biodiversity is to provide a standardized view of these heterogeneous data sources to improve interoperability. Fundamental to this advance are definitions of common terms. This paper describes the evolution and development of Darwin Core, a data standard for publishing and integrating biodiversity information. We focus on the categories of terms that define the standard, differences between simple and relational Darwin Core, how the standard has been implemented, and the community processes that are essential for maintenance and growth of the standard. We present case-study extensions of the Darwin Core into new research communities, including metagenomics and genetic resources. We close by showing how Darwin Core records are integrated to create new knowledge products documenting species distributions and changes due to environmental perturbations.

## Introduction

Concern over global loss of biodiversity [1]–[5] has resulted in widespread demand for quick, reliable access to high-quality data on the spatio-temporal occurrence of species and their relation to the environment. Initial studies have documented the response by species to diverse agents of environmental change [6]–[7], including alarming vertebrate extinctions [8]. The signature of climate and other environmental changes and their effects on biodiversity is now well documented, and the evidence is overwhelming [9]–[14].

The documentation of global patterns of biodiversity change is an important first step toward a wise, sustainable policy of conservation and management in the face of a changing environment [15]. Such documentation has emerged as a global priority [16]–[18]. A major impediment to creating this documentation is the lack of easily accessible data at the scope and at the scales needed. Most studies documenting biodiversity changes are limited to a few well-studied species or to small temporal and spatial scales [19]. To make effective use of existing data for broad-scale analyses, community coordination is required. Information must be in digital form, accessible, discoverable, and integrated. Each of these criteria presents distinct challenges, some of which are unique to biodiversity-related data.

A general challenge of biodiversity data that is shared with many other information domains is the lack of a coordinated publishing and integration system; data repositories tend to be isolated from each other in the absence of standards for data and communication protocols. Heterogeneity in meaning and content of terms creates obstacles in every aspect of data integration and use, including discovery, comparison, and quality assessment. Information communities, especially Library Information Sciences, have a long history of meeting these challenges through the creation and maintenance of standards.

**Figure 1:**
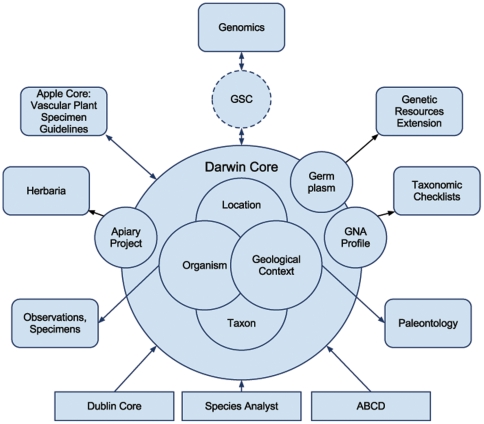
Scope of Darwin Core: The Standard, deriving from previous standards work (e.g., Dublin Core), describes core sets (e.g., organismal, taxonomic) of characteristics of biodiversity, which are applicable in many biological domains (e.g., Paleontology, Botany). The standard can be extended to cover details of specific sub-disciplines (e.g., Genetic Resources, Herbaria, Taxonomic Checklists). Collaborations with other standards organizations (Genomics Standards Consortium (GSC) extend Darwin Core for new disciplines (Genomics, Metagenomics, Gene Marker Sequences.

Darwin Core [20] is a standard for sharing data about biodiversity – the occurrence of life on earth and its associations with the environment. Darwin Core first emerged around 1999 as a loosely defined set of terms, and progressed through several iterations by different groups resulting in many different variants [21]. A formal set of terms and processes to manage changes were necessary to ensure utility for data integration. These aspects were developed within the Darwin Core Task Group [22] of the Taxonomic Databases Working Group (TDWG; www.tdwg.org) and ratified as a standard in October 2009. The philosophy for Darwin Core development has been to keep the standard as simple and open as possible and to develop terms only when there is shared demand. Darwin Core has a relatively long history of community development and is deployed widely [23]–[24]. For example, the Global Biodiversity Information Facility currently indexes approximately 300 million Darwin Core-formatted records published by more than 340 organizations in 43 countries. Increasingly, Darwin Core is being incorporated in communities beyond that of natural history collections (Figure 1), in which the standard has its roots.

In this paper, we describe the Darwin Core standard, its history, status, relationship to other standards, and prospects for further evolution. We also discuss how data sets are brought into Darwin Core compliance and how Darwin Core records are currently shared in a distributed publishing platform. We will demonstrate how Darwin Core continues to extend beyond its original formulation for natural history collections and will present, via use cases, how the standard continues to be reshaped and extended through new collaborations. We close by discussing innovative computational and informatics approaches to improving the scale, scope, and usability of networks that rely on Darwin Core compliant records.

## Methods

### Defining Darwin Core

The primary purpose of Darwin Core is to create a common language for sharing biodiversity data that is complementary to and reuses metadata standards from other domains wherever possible. Creating this common language has been particularly challenging, since natural history data curation practices have been developed locally and organically over hundreds of years, have varied between disciplines as well as institutions, and have had limited culture of data sharing.

**Figure 2:**
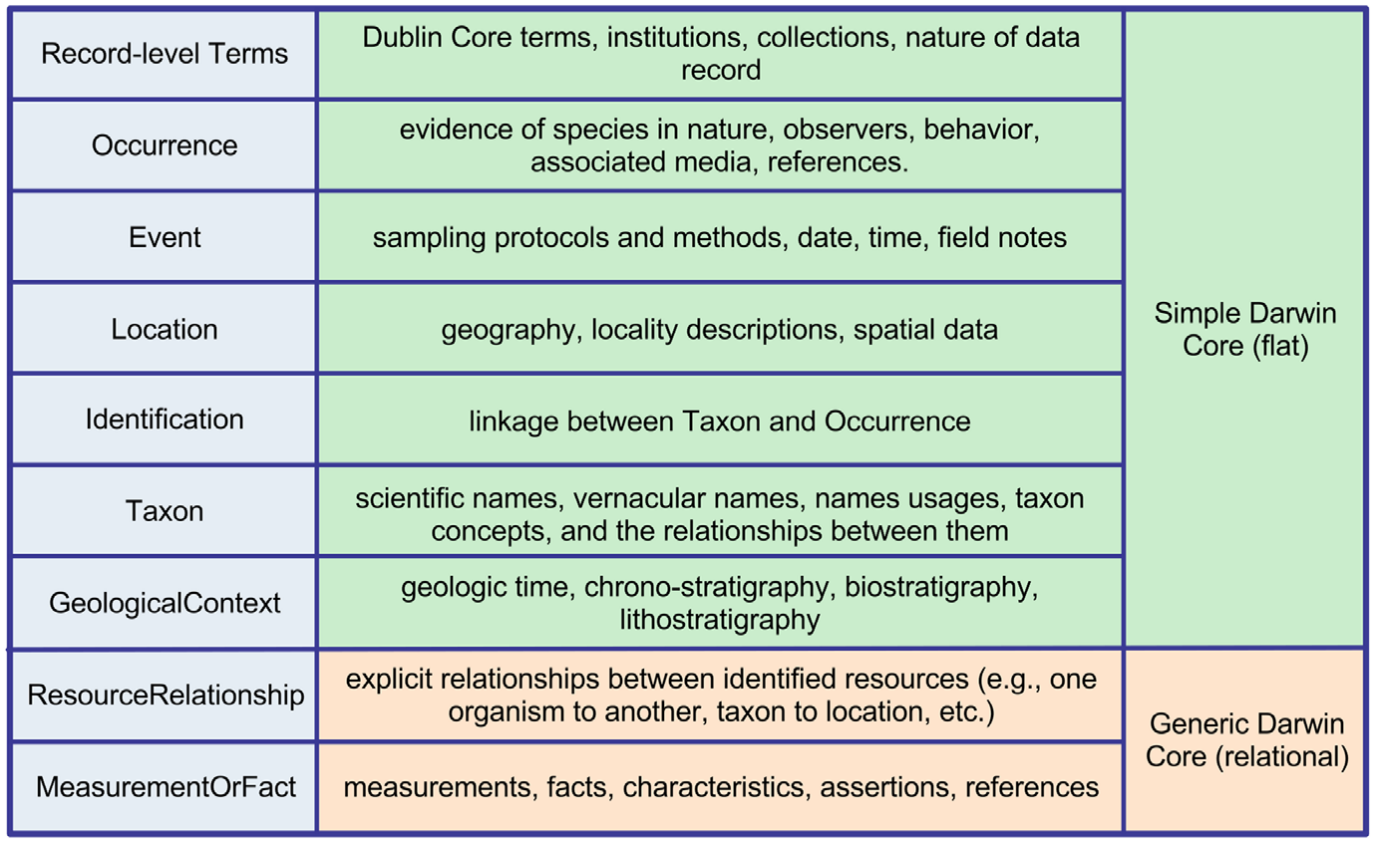
Darwin Core Categories: Simple Darwin Core is comprised of seven categories of terms (green). This subset of Darwin Core terms represents descriptive data about organisms that can be represented in one file with one row per record and one column per term. Two additional categories (orange) expand Darwin Core with concepts that require a more complex data structure, such as multiple measurements from a single specimen, and cannot be represented easily in Simple Darwin Core.

Fundamentally, Darwin Core is a set of terms having clearly defined semantics that can be understood by people or interpreted by machines, making it possible to determine appropriate uses of the data encoded therein. The terms are organized into nine categories (often referred to as “classes”, Figure 2), six of which cover broad aspects (event, location, geological context, occurrence, taxon, and identification) of the biodiversity domain. The remaining categories cover relationships to other resources, measurements, and generic information about records. Especially for the record level, Darwin Core recommends the use of a number of terms from Dublin Core (type, modified, language, rights, rightsHolder, accessRights, bibliographicCitation, references). The full set of current Darwin Core terms with their descriptions is available in the Quick Reference Guide (http://rs.tdwg.org/dwc/terms/).

The authoritative form of the Darwin Core is a downloadable archive [25], which contains various documents that are also available via web pages (http://rs.tdwg.org/dwc/). Key among the documents is http://rs.tdwg.org/dwc/rdf/dwctermshistory.rdf. This document includes descriptions of all terms, their histories, the relationships among them, and the relations between them and terms from other standards, all in a single file. The document is written in the Resource Description Framework (RDF) format, which is a standard for encoding the semantic structure of web-based resources. The same information is rendered in human-readable form on web pages (Quick Reference Guide, Type Vocabulary, Complete History, Mapping to ABCD, and Mapping to Old Versions). The standard also includes recommendations for implementation (Introduction, Simple Darwin Core, XML Guide, and Text Guide), a document describing how the standard is to be maintained (Namespace Policy), and a document describing the rationale behind any changes that are made to the standard (Decision History). In addition to the authoritative standard, Darwin Core consists of non-normative documents, commentaries, and tools tracked separately (http://code.google.com/p/darwincore/).

Though the scope and governance of Darwin Core are distinct from Dublin Core [26], readers will recognize the ancestry of Darwin Core in their shared traits: mission, principles of operation, and inherited terms. Indeed, if words were nucleotides, the Darwin Core mission is the same as that of Dublin Core except for one base-pair addition (in bold), namely, “to provide simple standards to facilitate the finding, sharing and management of **biodiversity** information.” This relationship is not accidental. The Dublin Core Metadata Initiative (DCMI) has done a very good job of maintaining the metadata specifications at the core of web-based resources. The success of DCMI encouraged TDWG to adopt similar policies for the specific domain of biodiversity through the Darwin Core. This process, in turn, acts as the basis for the development of data standards for even more specific sub-disciplines (e.g., plant genetic resources) as well as a basis for collaboration with complementary related domains (e.g., metagenomics).

### Implementation Guidelines

TDWG, as with other information standards bodies, has a history of developing data sharing standards that are bound to specific technologies, such as eXtensible Markup Language (XML). While Darwin Core is currently maintained in RDF, the enduring value of the standard lies in simple definitions of terms and their relationships to other terms, independent of technical implementation. As with Dublin Core, the idea is to promote use of the common terms in every appropriate context, and to leave the implementation details to specific applications. Data can be shared using Darwin Core in a variety of encoding schemes (Comma Separated Values, XML, JavaScript Object Notation, RDF, etc.). To aid in using Darwin Core in different contexts, there are documents with recommendations on how to share information as Darwin Core using various technologies.

The Simple Darwin Core (http://rs.tdwg.org/dwc/terms/simple/index.htm) document explains the capacity to share records with properties that do not repeat, as simple text files or XML. There is also an XML Guide with reference XML schemas for highly structured data and a Text Guide explaining the construction of Darwin Core Archives (a combination of CSV files and a simple XML document describing the semantics of the data file columns and their relationships to each other). Darwin Core Archives can support structured data that conform to a star schema (a single core set of records in one or more files, with one-to-one or one-to-many relationships to records in other files). The Darwin Core Archive is becoming ever more popular as an easy means for data sharing, largely due to the release of supporting tools, in particular the next-generation data publishing system – the Integrated Publishing Toolkit (http://www.gbif.org/informatics/infrastructure/publishing) – an open source application developed by the Global Biodiversity Information Facility discussed in further detail below. Darwin Core Archives have been adopted by Pensoft® (http://www.pensoft.net/journals/) as the preferred form for data appendices to taxonomic publications in MycoKeys, PhytoKeys, and ZooKeys.

## Results

### Development and Ratification as a Standard

Darwin Core's development spans more than a decade, but rests on the earlier work of academic societies that addressed how to document or catalog specimens in natural history collections. Those societies put forward prescriptive guidelines about how to catalog specimens properly, first on paper and later in computer databases [27]–[29]. The purpose was to promote completeness in capture and consistency in representation or encoding. Actual data exchange and integration was anticipated as a future capability, but these guidelines preceded the Internet and World Wide Web.

In the early 1990's, entity-relationship modeling had become accepted as a prerequisite for designing any database serving multiple users. A normalized database structure was the primary technique for enforcing data integrity. The Association of Systematic Collections (ASC) model [30] was the result of the first group effort to create a conceptual model for biological collections that accommodated the distinct taxonomic disciplines commonly found in natural history museums. The ASC model was an ontological description of the domain, but was determined at the time to be beyond the technical capabilities to implement [31]. Eventually, this model was then used as the point of departure for several collection management applications, including Specify (http://specifysoftware.org/), Biota (http://viceroy.eeb.uconn.edu/biota), and Arctos (http://arctos.database.museum/). The emphasis at that time was to develop best practice for data capture and management, not data exchange or integration.

The first successful tool to enable data integration in the biodiversity domain came to be known as the Species Analyst. A prototype based on the Z39.50 protocol [32], which was popular in the Library Science community at that time, was deployed in a pilot project, the Z39.50 Biology Implementers Group (ZBIG, U.S. National Science Foundation NSF-DBI-9811443). An outcome of the first meeting of that project was a list of terms with definitions to be included in the profile. The name “Darwin Core” was coined by Allen Allison and adopted at that meeting. The Species Analyst, a project developed under the NSF-funded ZBIG project at the University of Kansas [33], led to an international deployment of data servers [34] for ornithological (NSF-DBI-9808739) and ichthyological (NSF-DEB-9985737) [35] collections.

The initial purpose of the Darwin Core was to facilitate the exchange of information about the geographic and temporal occurrence of organisms in the natural world and the physical existence of specimens in biological collections. In contrast to earlier guidelines, Darwin Core was not a prescription for how to manage collection information. Instead, it was designed to circumscribe a conceptual model for a research community aiming to create a loose federation of databases and advance its research capabilities through data integration. The barriers to publishing data in Darwin Core were purposefully kept as low as possible.

The informal Darwin Core terms developed for Species Analyst were further refined under the NSF-funded Mammal Networked Information System project (MaNIS, NSF-DBI-0108161, http://manisnet.org) [36] and the Ornithological Information System project (ORNIS NSF-DBI-0345448, http://ornisnet.org) [37]. Major goals of the MaNIS project were to employ HTTP as the transport protocol instead of the less widely used Z39.50, to create a message protocol to support web-based distributed queries (Distributed Generic Information Retrieval – DiGIR), and to enhance the set of terms to include information such as georeferences based on guidelines that address the capture of data quality metrics [38]. The goal under ORNIS was to make sure that the Darwin Core underwent the process of becoming a formal standard.

Darwin Core was developed in parallel with efforts in Europe to establish a comprehensive model for biological collections information — Access to Biological Collections Data (ABCD) [39]. The philosophies of the two approaches were distinct, even if they both had the goal of promoting sharing of biodiversity information. Darwin Core was designed to be minimal (only terms shared in common throughout natural history collections) and flat (no relational structure), while ABCD was highly structured and sought to capture the wide variety of biodiversity data and their relationships. Both groups defined their data models in XML schemas. ABCD was ratified as a standard by TDWG in 2005. Darwin Core departed from defining the standard in XML schema and followed the DCMI model, incorporating the experience of the previous decade to establish a set of terms in wide use in practice for data sharing.

Prior to ratification, Darwin Core had a history of versions [21], each attempting to adapt to expanding community-defined needs. The earliest versions were limited to defining terms for biological specimens in collections. Continued development in sectors of the community produced versions with added capabilities targeting observations [40] and paleontology [41]. Standard Darwin Core reconciles the omissions from, incompatibilities between, and inconsistencies within previous versions and provides mappings to the out-dated terms as well as mappings to concepts in the ABCD Schema. In addition, terms were added to enable the transmission of taxonomic name and classification data sets. The result is a relatively simple and stable specification, which serves the needs of data publishers, data consumers, and application developers to work with primary biodiversity data.

Darwin Core underwent a year of document development by the Darwin Core Task Group with support from the National Science Foundation and the Global Biodiversity Information Facility. The draft standard underwent review with public commentary before being ratified by the TDWG Executive Committee in October 2009.

### Maintenance and Evolution

In combination with openness and consensus building (key aspects of the TDWG constitution: http://www.tdwg.org/about-tdwg/constitution/), two of the guiding principles behind Darwin Core are flexibility and adaptability. The standard has to be flexible to accommodate a variety of ever evolving technical contexts in which it might be used (spreadsheet columns, relational database fields, XML schemas and documents, non-SQL data stores, and semantic web resources). The standard has to be adaptable to accommodate growth (new terms, additional meanings) and interoperability (connections to related information). To thrive, the standard must have a means to adapt to changing needs.

Darwin Core development is community driven and follows the process published in the Darwin Core Namespace Policy (http://rs.tdwg.org/dwc/2009-09-23/terms/namespace/index.htm). Open commentaries on issues related to Darwin Core are expressed through a public forum (at tdwg-content@lists.tdwg.org) to which any subscriber may contribute. Discussions that result in consensus for change (new terms, new definitions, new relationships, changes in documentation) are submitted as change requests using templates requesting the appropriate information on the issue tracker of the Darwin Core Project site (http://code.google.com/p/darwincore/wiki/SubmittingIssues). Sometimes this process is reversed, with a submitted issue being copied to the discussion forum for elaboration and consensus before refining the issue as submitted. All changes undergo a 30-day public review, announced on the listserv. Changes are reviewed by TDWG's Technical Architecture Group (TAG: http://www.tdwg.org/activities/tag/), a set of volunteer members with broad interest and expertise in web-based interoperability. The TAG determines if the proposed changes are compatible with Darwin Core and that they do not reinvent existing standards. Changes that pass this review process are made as version changes to the affected documents, the latest of which always constitute the current standard.

### Data Quality and Constraining Darwin Core

Data quality and fitness for use are primary concerns in the biodiversity community [42], where information comes from heterogeneous sources spanning the globe over hundreds of years. Both data discovery and data quality assessment are hampered by this heterogeneity. Part of the solution depends on the use of common vocabularies, part on dictionaries of synonymous terms, and a great deal on applications that can facilitate discovery and assessment in place of or to aid in otherwise labor-intensive “cleanups”. For example, having a dictionary that equates “voucher” and “sample” with an accepted term “PreservedSpecimen” would allow applications to make this substitution automatically and allow users to discover relevant data using any of the synonyms regardless of how they were stored in the original data.

Darwin Core makes recommendations about constraints on data content, whether about data types, valid values, or controlled vocabularies. For example, decimalLatitude is recommended to be a floating-point number falling between 90 and −90, inclusive; countryCode is recommended to be a value from ISO 3166-1-alpha-2 (two-letter country codes). Though these recommendations are made in the standard, the philosophy is to relegate enforcement to applications where they make sense. For example, in a collaboration where the purpose is to automate data quality improvement on shared data, it is essential to be able to share the warts-and-all data that need to be investigated and improved.

## Discussion

### Frontiers

Though the Darwin Core is defined in an RDF document, integration of biodiversity data in the semantic web is in its early stages. One of the major challenges for Darwin Core in the semantic web context is the lack of a well-defined ontology - a formal definition of relationships between terms in a defined domain. An ontology would define the relationships between concepts such as biological entities, the events that document where and when they occurred, and the processes through which they are identified as being representative of a taxonomic concept. Without rigorous relationships between concepts and the properties that define them, connections between biodiversity data and related semantically rich information, such as literature and genomes, are difficult to traverse. This creates obstacles to cross-disciplinary semantic inquiry, such as in the Linked Data distributed data community (http://linkeddata.org/). Current research is trying to address this gap (see TaxonConcept Knowledge Base, http://www.taxonconcept.org/ and BiSciCol, http://biscicol.blogspot.com/), active discussions on various aspects of the challenge take place regularly on the Darwin Core public forum (http://lists.tdwg.org/mailman/listinfo/tdwg-content), and an RDF task group has been established within TDWG “to adapt TDWG vocabularies for use as RDF classes and properties and to integrate those resources with other well-known vocabularies and ontologies outside TDWG for use in describing biodiversity resources.” [43]

### Tools and Infrastructure for Publishing Darwin Core Data

A variety of tools has been developed to simplify the mobilization and sharing of biodiversity data using the Darwin Core. Many of these tools focus on the creation or validation of Darwin Core Archives (http://code.google.com/p/darwincore/wiki/ToolsAndApplications#Tools). The Global Biodiversity Information Facility (GBIF), an international organization promoting the free and open access to biodiversity data online, has invested heavily in the open and collaborative development of tools (http://tools.gbif.org/) to make the creation and publication of Darwin Core Archives relatively simple.

The Integrated Publishing Toolkit (IPT; http://www.gbif.org/informatics/infrastructure/publishing/) is an open-source software development project, led by GBIF, designed to address the challenges of sharing biodiversity data. By producing and providing easy access to Darwin Core Archives and resource metadata in Ecological Markup Language [44], the IPT works in tandem with a Global Biodiversity Resources Discovery System (GBRDS), a data registry, to facilitate discovery and retrieval of shared data throughout the world. The IPT complements other tools that have been created to assist data users to explore, organize, and mobilize data sets. A range of spreadsheet and archive creators and validators has been developed by GBIF and others. These tools can be accessed via the GBIF Publishing Software web page (http://www.gbif.org/informatics/standards-and-tools/publishing-data/publishing-software/).

Whereas tools such as the IPT facilitate data mobilization using Darwin Core data, other tools build on these sources to integrate and index biodiversity data. These aggregators are thematic networks with either geographic (e.g., Atlas of Living Australia, http://www.ala.org.au/) or taxonomic focus (e.g., VertNet, http://vertnet.org). Developing these systems to work sustainably and at large scales is an area of active research. For example, VertNet (http://vertnet.org/), an aggregation of vertebrate specimen and observation data into a cloud-based data store, makes use of the Simple Darwin Core in comma separated value (.csv) files with header rows containing Darwin Core term names. These files are published to the data store where the records are indexed for high-performance querying.

### Extensions

Because Darwin Core aims to cover the common ground in biodiversity, it inevitably lacks terms that are of interest to more specialized groups. A Darwin Core Extension consists of additional terms describing a complementary, related domain, or guidance on the use of Darwin Core within a specific sub-domain of biodiversity. Over the last decade, Darwin Core has evolved by means of extensions, some of which have been incorporated in the standard. For example, the terms of a paleontology extension related to an earlier, pre-standard version of Darwin Core became the terms of the current GeologicalContext class. Similarly, terms originating from an early version of the Darwin Core created for the Ocean Biogeographic Information System (OBIS; http://www.iobis.org/) have also been added to Darwin Core. Going forward, specific projects or disciplines may find it convenient to extend the scope of Darwin Core outside the existing namespace and governance process for proof-of-concept research. Once the understanding of the new terms has been tested and proven of utility in a broader context, incorporation into the standard can be accomplished through community consensus.

### Integration of Darwin Core with New Research Communities

Darwin Core continues to be integrated into new research communities. For example, the Nordic Genetic Resource Center (NordGen; http://www.nordgen.org) and Bioversity International (formerly IPGRI) (http://www.bioversityinternational.org) have sought to take advantage of Darwin Core on behalf of the European plant genetic resources and genebank community and, at the same time, share rich discipline-specific genetic resource information. A careful review was made of Darwin Core and a set of additional terms that did not overlap or conflict with existing Darwin Core terms was proposed to meet the community's needs. The new terms, modeled directly from FAO/IPGRI Multi-Crop Passport Descriptors [45] (2001) and the proposed EPGRIS3 trait data standard for characterization and evaluation data [46], were expressed and published openly in an XML schema for germplasm data (DwC-germplasm, http://code.google.com/p/darwincore-germplasm/) [47], resulting in the first successfully deployed extension to the Darwin Core Standard.

The success of DwC-germplasm has encouraged other communities to supplement or work in parallel with Darwin Core to meet specific needs. For example, the Apiary Project (http://www.apiaryproject.org/) extended the Darwin Core to capture basic specimen information along with detailed annotation data (e.g., annotatedBy, dateAnnotated) found on herbarium sheets. Other communities are focusing on providing guidelines on how to use Darwin Core within a discipline without the need for extensions. The Apple Core Initiative (http://code.google.com/p/applecore/wiki/Introduction), organized by Canadensys, (http://www.canadensys.net/), is aimed at providing guidance on best practices for the content of Darwin Core terms for vascular plant specimens.

A set of taxonomic extensions collectively known as the “GNA Profile” (named for the Global Names Architecture) [48]–[49] have been designed by GBIF with input from Catalogue of Life (http://www.catalogueoflife.org/), Encyclopedia of Life (http://eol.org/), nomenclators, and other taxonomic initiatives. These extensions (http://rs.gbif.org/extension/gbif/1.0/), together with the taxonomic terms of Darwin Core, allow sharing of rich taxonomic data often found in species checklists. The extensions are designed to be used in a one-to-many relationship with core taxon records, allowing the sharing of structured data such as vernacular names, species range distributions, textual descriptions, type species and specimens, image data, and bibliographies.

An effort is underway within TDWG to create a standard for biodiversity multimedia resources and collections, called the Audubon Core [50]. The proposal introduces vocabularies covering the management and content of biodiversity-related media and their taxonomic, geographic, and temporal scope. Audubon Core adopts by inclusion a number of terms from Darwin Core, which can reduce the burden on existing media publishers who may have already used Darwin Core terms for describing the media content.

In collaboration with the Genomic Standards Consortium (GSC; http://gensc.org/) [51], Darwin Core is now being extended to cover DNA-level observations (genomes, metagenomes, and gene marker sequences). The GSC, a standards body similar in scope to Biodiversity Information Standards (TDWG), is working to “standardize the description of genomic data and promote the exchange and integration of genomic data. [52].” Advances in technology have made sequence-based diversity assessments increasingly routine in biodiversity research. The GSC has developed the MIxS (Minimum Information about any (x) Sequence) standard containing checklists for describing genomes, metagenomes, and marker gene studies. Conceptually this approach is similar to Darwin Core in that it defines terms for the description of data, but differs in that it requires the reporting a certain minimum set of descriptors such as environmental and geographic origin and details about sample and sequencing processing in addition to any kind of genomic sequence.

Currently, Darwin Core and GSC developers are collaborating (supported by NSF-RCN4GSC) to harmonize Darwin Core and MIxS to aid the growing field of sequence-based diversity research. Darwin Core and GSC developers have concluded that the standards are conceptually similar and that the distinct sets of terms are complementary. This allows the development of a bi-directional (see Figure 2) solution, where in the future the GSC will be able to incorporate terms from Darwin Core and add GSC-specific reporting requirements, while Darwin Core will be able to offer a genomics extension using the results of this activity.

### Scope and Scale of Darwin Core for Biodiversity Science

The creation of a standard format for biodiversity data, the development of aggregation tools, and the increasing use of Darwin Core Archives have helped to overcome the challenge of limited availability of data to answer questions about biodiversity. As more data become digitized and discoverable, biocollections can become a window into broader analyses of ecological, climate, niche, environmental, and biological research questions and critical issues.

Though predated by examples of the effective use of Darwin Core for distribution modeling, influence of natural area preservation, and estimates of climate change impact [53], the utility of data shared using Darwin Core is illustrated by two recent examples. First, the University of California, Berkeley, and the Wildlife Conservation Society have collaborated to create Réseau de la Biodiversité de Madagascar (REBIOMA, http://www.rebioma.net/).The effort seeks to make biodiversity data about Madagascar available via a single web-based resource. Primary data on biodiversity in Madagascar come from researchers and institutions all over the world. REBIOMA works with each collection to standardize the data using the Darwin Core, to mobilize and aggregate the data, and to vet the data for quality, completeness, and fitness for use. Data passing automated quality checks and review by a board of taxonomic experts are analyzed in combination with environmental data to produce maps showing models of where species might occur. The success of this project has made it possible for researchers, policy makers, government officials, and conservationists to capture a wealth of data to address issues of Malagasy biodiversity. The simplicity and flexibility of Darwin Core has made it possible for REBIOMA to provide immediate access to high-quality data and tools for monitoring and assessing conservation efforts.

A second example of innovative use of Darwin Core data is the Map of Life Project (http://www.mappinglife.org/), and sister projects such as LifeMapper (http://www.lifemapper.org/) [54], which aggregate and mobilize sources of species distribution information and provide tools to visualize and analyses those data. In Map of Life, species occurrence data points formatted as Darwin Core are queried and brought into the application with related data sources such as range maps, assemblage checklists, and habitat preference data. Integration of these data types and modeling will lead to summary products describing species distributions and provide the basis for distribution change assessments in the face of human-induced global changes [55]. The Map of Life will be a knowledge base of hundreds of thousands of high-resolution species distribution maps covering a wide range of taxa and geographic locations.

### Conclusions

The demand for Darwin Core data is increasing. The corpus of information spanning three centuries of biodiversity exploration is being digitized at an increasing rate. Major efforts such as the new US National Science Foundation program Advancing Digitization of Biological Collections (http://nsf.gov/funding/pgm_summ.jsp?pims_id=503559&org=DBI&from=home) and the Atlas of Living Australia (http://www.ala.org.au) are two recent examples. A flood of qualitatively new global biodiversity data is also being generated through the application of high-throughput gene sequencing of environmental samples.

The President's Council of Advisors on Science and Technology (PCAST) report on sustaining environmental capital noted recently that the challenge is not only one of the volume of data, but also its heterogeneity, as traditional biotic surveying practices blend with new approaches, such as remote sensing [56] or metagenomics [57]. A key passage in that PCAST report captures the challenge perfectly: “temporal, spatial, and methodological heterogeneity leads to a depth and richness in biodiversity and ecosystems data not found in other fields. It also makes dataset interoperability a problem that is at the same time particularly challenging and highly important to solve [58].” Darwin Core fills an essential role in describing some of these data in a standard format based on community input and demonstrated utility.

Darwin Core is a living standard. Although its roots were planted in the vertebrate natural history collections community, Darwin Core continues to grow to serve the needs of biodiversity research. This growth, over the relatively short period of a decade, speaks both to the need for such a standard and to the efforts of its champions to increase its utility through a community development process in order to meet increasing demand. Darwin Core greatly increases the value and re-use of freely available and accessible biodiversity data so that they can be effectively mobilized, integrated, and incorporated into other “grand challenge” scientific endeavors.
